# Managers’ experiences of implementing palliative care in nursing homes: a qualitative follow-up study after an educational intervention for the staff

**DOI:** 10.1186/s12904-026-02276-w

**Published:** 2026-08-11

**Authors:** Helene Åvik Persson, Anna Sandgren, Gerd Ahlström

**Affiliations:** 1 Department of Health Sciences, Faculty of Medicine, Lund University, BMC E15, Box 117, Lund, 221 00 Sweden; 2 Center for Collaborative Palliative Care, Department of Health and Caring Sciences, Faculty of Health and Life Sciences, Linnaeus University, Växjö, Sweden

**Keywords:** Educational intervention, Implementation, Managers, Nursing homes, Palliative care

## Abstract

**Background:**

It is crucial to implement knowledge-based, high-quality palliative care for the increasing number of frail older people with multimorbidities in nursing homes. The manager’s role in a successful implementation is often taken for granted despite scarce evidence. Therefore, the aim of this long-term follow-up study was to explore how managers experienced the implementation of palliative care in nursing homes after a multicentre educational intervention for staff. This Implementation of Knowledge-Based Palliative Care in Nursing Homes (KUPA) program consisted of workplace seminars, reflective activities, and practice-based tasks between seminars. The implementation was research-led before the participating managers took over its governance.

**Methods:**

This qualitative follow-up study considered semi-structured interviews with 22 managers responsible for implementing palliative care in 20 nursing homes in Sweden. The analysis was conducted using a reflexive thematic approach.

**Results:**

Our findings are presented under three main themes: Navigating Organisational Barriers, Empowering the Action Plan, and Ripple Benefits of Education. The managers *navigate organisational barriers* by establishing stability, maintaining continuity and supporting staff members’ knowledge development despite structural barriers and limited resources. *Empowering the action plan* meant supporting staff to maintain motivation and a positive attitude towards workplace change. Managers emphasised their responsibility for the continuation of implementation of palliative care. The *ripple benefits* *of* *education*, from the managers’ perspective, include increased knowledge and use of holistic palliative care, a shared language, and a consensus on the meaning of palliative care, thereby expanding cooperation and mutual understanding across the team’s professions.

**Conclusions:**

This long-term follow-up study revealed several challenges to the implementation of palliative care and underscored the demanding work of managers. Staff shortage, limited budget and support led to work-related instability. Closer interprofessional collaboration and a broader approach to palliative care as a process from early signs to the end of life need to continue and deepen to be knowledge-based nursing care. From a leadership perspective, a successful implementation requires a long-term commitment, financial resources, a supportive organisation, and manager competence.

**Trial registration:**

NCT02708498. Date of registration: 26 February 2016.

**Supplementary Information:**

The online version contains supplementary material available at 10.1186/s12904-026-02276-w.

## Introduction

Demographic shifts across Europe, characterised by the increasing older population, have heightened the demand for high-quality palliative care services in nursing and care homes [1, 2]. Such care facilities provide accommodation and services for frail older people, who are among the most vulnerable members of society. These older residents often have multiple morbidities, including cancer, cardiovascular diseases and dementia, and are in need of palliative care adapted to their conditions and the progression of their illnesses [1, 3, 4]. Most studies on palliative care improvement have been conducted in hospital settings and have focused on nurses and physicians [5]. The limited knowledge regarding the implementation of palliative care for older people with multimorbidities [2, 6], requiring long-term care assistance, indicates the need for a greater focus on palliative care education for nursing home staff [1, 3–5].

Multi-centre studies reported that nursing home staff’s agreement with the principles of general palliative care varied across several countries, indicating a clear need for improvement [7, 8]. These studies were included in the PACE project, a European project investigating palliative care in nursing homes across six countries, namely, Belgium, England, Italy, Finland, the Netherlands, and Poland [8]. In Swedish nursing homes, where nurse assistants and care assistants provide most of the daily nursing care, experience-based care is commonplace. Consequently, knowledge of palliative care is limited [7, 8, 9]. To address this research gap, we initiated a project called Implementation of Knowledge-Based Palliative Care in Nursing Homes (abbreviated “KUPA”) as an educational intervention for staff [10, 11].

The literature on building palliative care capacity in nursing homes has highlighted the need for engagement of leadership for successful implementation [10, 12–15]. Nevertheless, there has been little empirical research on leadership’s role, particularly in the field of implementation of evidence- or knowledge-based palliative care in nursing homes [10, 16]. Håkansson et al. [16] reported that a limited knowledge of palliative care and a lack of supportive organisation with clear expectations and goals hindered managers’ ability to take on full leadership responsibility for implementing palliative care in their nursing homes. In the KUPA project, managers’ views were explored through the lens of the theory of organisational readiness early during staff education [10, 11]. The main barriers to organisational readiness reported by managers were a lack of trust in available time and resources for implementation. Managers felt that staff members lacked confidence in their ability to face dying older people and had negative attitudes towards changes in their daily work as well as in care routines [10]. The aim of this long-term follow-up study of the KUPA project was to explore how managers experienced the implementation of palliative care in nursing homes after an educational intervention for staff.

## Methods

We conducted this follow-up study 1.5 years after the initiation of the KUPA educational intervention for staff. The study employed an explorative qualitative design based on semi-structured interviews with the responsible first-line managers taking part in palliative care education. We used the reflexive thematic analysis approach of Braun and Clarke [17, 18], which incorporates the analysts’ reflections of their backgrounds and preconceptions into theme construction. This study was conducted and reports results in accordance with the Consolidated Criteria for Reporting Qualitative Research (COREQ) checklist [19].

### Nursing home setting

Older people in Sweden who became too ill or frail for living at home move to a nursing home [20]. Nursing homes provide a home environment in which each person leases a small private apartment, typically consisting of one room, a small kitchen area and an accessible bathroom. Several apartments are clustered in a single building, which includes a large communal area. The eligibility criteria for being allocated an apartment in a nursing home are strict; only those who require extensive care and support are eligible [21]. Onsite care is provided 24 h a day, 7 days a week, and is regulated by two laws: the Social Services Act [22] and the Health and Medical Services Act [23]. The former act governs assistance with daily activities, such as dressing, showering, cleaning, meals, indoor and outdoor activities, and the encouragement of social interactions, which care assistants and nurse assistants provide. The latter act regulates health interventions, including medications and wound dressing, which are provided by onsite or offsite registered nurses, and medical examinations and prescriptions are provided by offsite general practitioners employed in primary health care (part of the county council). The Swedish National Board of Health and Welfare set the goal that all health care workers provide knowledge-based palliative care [24], and the Social Services Act has recently set the same goal regarding evidence-based social services [22].

### Research setting and educational intervention

This study is part of the KUPA project, which examines an implementation strategy consisting of a six-month educational intervention for nursing home staff conducted in 20 nursing homes. One inclusion criterion was that both smaller and larger nursing homes from rural and urban areas across two counties were included to reach satisfactory transferability of the results [25]. Additional inclusion criteria were that first-line managers of the nursing home participate in training led by researchers and agree to continue serving as responsible for education in the second phase until all staff, including new recruits, are trained.

The included nursing homes were a mix of larger (≥ 100 older people) and smaller (< 25 older people) nursing homes in both urban and rural settings across two counties. The evaluation of the implementation strategy in the KUPA project used a non-randomised experimental design with intervention and control groups and pre- and post-assessment [11]. The research design, methods and substudies are described in detail in the published study protocol [11].

The researchers developed the KUPA educational content and guide booklet through discussions with staff, informal caregivers, and patients representing both hospital and community care [11]. Thereafter, we completed a pilot test of the educational intervention at four nursing homes using the educational booklet as a study material for the participants [11]. The education is described in Supplementary Material 1 (attached before the reference list). Table 1 provides an overview. The educational intervention consisted of five seminars lasting three hours each. Each seminar group consisted of 8–10 nursing home staff members, including the first-line manager. The seminars focused on knowledge-based palliative care according to research- and best practice-based knowledge. They involved reflective practice activities [26] along with lectures on palliative care, films and anonymised cases from these nursing care practices. The booklet covered the five seminars’ themes, a reference list for self-study, a set of individual and group tasks for each seminar, and tasks to be completed between seminars [11, 27]. By bringing own daily work into the seminar discussions, participants adapted their new knowledge of palliative care to the context of their respective nursing homes.

**Table 1:** Content of the five seminars published in the study protocol [11]

	Theme	Content
Seminar 1	The palliative approach and dignified care	The purpose of this theme is to increase knowledge and understanding of the values on which palliative care is based, its ethics, and its approach to the patient.
Seminar 2	Next of kin	The purpose of this theme is to increase knowledge and understanding of the emotional context and the role of the next of kin and to consider how the family´s need for support can be better met.
Seminar 3	Existence and dying	The purpose of this theme is to increase awareness and understanding of one’s own thoughts concerning existence and death with dignity so that staff can feel secure during challenging conversations with patients and the next of kin.
Seminar 4	Symptom relief	The purpose of this theme is to increase understanding of the importance of effective symptom alleviation in palliative care and to promote the use of validated rating instruments.
Seminar 5	Collaborative care	The purpose of this theme is to foster a deeper understanding of the importance of teamwork, with a particular focus on care, implying collaboration between staff, the patient, and the next of kin.

### Participants in follow-up study

Participants were the 20 managers from each of the KUPA project nursing home and the 2 district managers who oversee these nursing homes in their respective counties [11]. The nursing home managers, except for five that were newly employed, had also participated in a previous interview for the KUPA study, approximately one year earlier [10]. One manager of those five, attended the interview accompanied by a registered nurse who had participated throughout the education at this nursing home.

### Interviews and data collection

Data collection in this study was performed 9–12 months after the researcher-led implementation of the KUPA project (March–May 2018). The interviews were conducted by the same two interviewers who led the previous interviews [10]. The interviewers had met the managers during the palliative care seminars; they both held doctoral degrees and had prior clinical experience in both palliative care and elderly care, but were not employed at any nursing homes. In preparation for data collection, the interviewers listened to the previous interview and constructed additional follow-up questions. This enabled them, during the interviews, to consider the conditions of each manager, given the specific nursing home’s context and organisation. The interviews were conducted in a separate room at the managers’ workplace and recorded digitally. The semi-structured interview guide consisted of two main questions: What does the educational intervention mean for palliative care in the nursing home(s) for which you are responsible? How does the implementation of palliative care continue after the project period? Follow-up questions were formulated on the basis of participants’ answers to the two main questions and the narratives from the previous interview. These questions were phrased as follows: Could you tell me more about this implementation? What do you think facilitated or hindered the implementation? The same questions were asked of the two top-level managers and the front-line managers to obtain a more in-depth view of the implementation of palliative care.

### Data analysis

We analysed the interview responses using an inductive approach, following the reflexive thematic analysis described by Braun and Clarke [17, 28], including repetitive engagement with the dataset. This method of analysis was chosen because it offered an opportunity to explore and interpret the data by identifying patterns of meaning in the collected data [16, 17]. First, two of the authors (HÅP, GA) read and listened to the interviews several times to become familiar with the data and identify any patterns or themes. Second, the analysts applied an inductive coding approach to each interview, with an iterative analysis of the transcripts performed, using a coding structure supported by the NVivo 15 software package [29]. Third, the creation of initial themes was facilitated. Patterns of meaning were developed by sorting codes into broad topic areas. During phases 4 and 5, these broad topic areas were subsequently refined in greater depth by defining and naming themes. Finally, phase 6 consisted of creating a written presentation of the results, which enabled a further engagement with the data and allowed a greater refinement of subthemes and themes. Throughout the entire analysis process, the two authors discussed progress with the second author until a consensus was reached.

## Results

All of the 22 participating managers were female except for one male; the ages of participants varied between 38 and 68 years, with a mean age of 54.8 years. The number of years in the managerial position ranged from 2 to 41 years, with a mean of 18.4 years. Managers’ educational backgrounds were as follows: social workers (*n* = 12), registered nurses (*n* = 5), human resources or behavioural education (*n* = 3), sociology (*n* = 1), and several types of education (*n* = 1). Interview duration averaged 46 min (range: 31–74 min).

The only main difference in the responses between the district and nursing home managers was that the former could attest to variations in the level of implementation across the nursing homes; therefore, the authors decided not to report their answers separately. A thematic analysis revealed three themes and seven subthemes (Table 2).

**Table 2:** Overview of themes and subthemes

Theme	Subthemes
Navigating organisational barriers	Handling structural obstacles Finding and retaining stable staff Loss of momentum
Empowering the action plan	Overcoming existing emotions Reflection and dialogue as motivational strategies
Ripple benefits of education	Understanding the changes to the palliative approach Cooperation between different professional groups

### Navigating organisational barriers

The managers emphasised the importance of establishing stability within the staff team, with such stability being essential for implementing palliative care. However, structural barriers and limited resources often hindered continuity and staff development. Furthermore, instability in the management and unclear responsibility for the continuation of implementation generated resignation and uncertainty among the managers. Together, these factors illustrate the managers’ barriers to ensuring knowledge-based palliative care for older seriously ill persons and their family members (Table 2).

#### Handling structural obstacles

The managers emphasised that various structural conditions, such as finances, time constraints, and top-down management, affected their ability to lead the implementation of palliative care in a long-term, high-quality manner. Feelings of limitations and powerlessness were often experienced when new directives from higher managerial levels arrived concerning the organisation’s budget. Financial constraints sometimes forced difficult choices as to priorities, with investments in skill development and staff support having to be postponed due to more urgent needs.

Sure, it´s a bit tight in a way, but it still feels like that the training is actually helping. We can´t cut back on everything. We can´t be ´stupidly stingy´. In the long run, I think that if we do this, we´ll end up with staff who feel secure in their jobs and know what they´re doing. I think it will reduce sick leave, for example. (Interview 10)

Despite these obstacles, the managers emphasised that palliative care was a matter close to their hearts and an area they believed had to be prioritised even when resources were scarce. They stressed the importance of staff daring to pause and reflect on what was truly important for the quality of care at the end of life. This concerned using available resources wisely and investing in older people’s dignity and quality of life.

And perhaps these days, when you have less money, you really have to sit down and ask yourself: what is important, what do we want to spend money on, or what should we spend money on? (Interview 2)

#### Finding and retaining staff

All managers experienced the challenges of recruiting and retaining staff at their nursing homes; notably, they explained this as a common issue in elderly care, with a strong impact on the implementation of new knowledge. They stressed that there was a significant shortage of nursing assistants, registered nurses and occupational therapists, which created a constant challenge in managers’ daily work and a barrier to implementing palliative care. The managers reported that it had become increasingly difficult to attract new employees, especially as the population aged and the need for comprehensive care grew.

Yes, it’s hard to find staff! Applications are not pouring in. If you look at how many unemployed people there are, applications are not pouring in if you compare them with the labour market figures. (Interview 16)

The managers who had taken over operations characterised by turbulence and high staff turnover described their difficulties in following the plan of the implementation. The focus had been primarily on creating basic stability rather than further implementing palliative care, and difficulties such as those in building a cohesive staff group that could work in the long term and implement and spread palliative care knowledge were highlighted. If professional categories among the staff were lacking, managers noted that the objective of the training could not be achieved, and such a lack hindered the implementation.

One obstacle I think of is if there is no nursing expertise… In those teams, not all professions are represented because there are gaps in the organisation; that could be an obstacle. (Interview 9)

#### Loss of momentum

The managers highlighted several reasons the implementation of palliative care had fallen by the wayside. Changes in management left new managers without necessary information about such an implementation, creating uncertainty about how to continue it. A strong sense of resignation and uncertainty was emphasised by those managers. This resulted in no clear action plan being established to continue implementing palliative care in the nursing home.

I feel a bit like I was thrown into it without any major information, so I probably didn’t really have any expectations. So, without that, I felt more that it’s an interesting topic. (Interview 12)

A few managers did not see the need to continue the implementation of palliative care in their nursing homes. A lack of time within the organisation and a lack of clarity about the manager’s responsibility for the continued implementation of palliative care further complicated the implementation process. Managers noted that the implementation of palliative care had paused after the final educational meeting, as no one took responsibility for continuing the process. As a result, plans for the implementation of the strategy at the nursing home were abandoned.

And I kind of feel like I have to address this. I have to but it’s hard… like… where is my responsibility in all of this? I know I have responsibility for my residents, but it (palliative care) is a health care responsibility. (Interview 19)

### Empowering the action plan

The managers considered the team’s openness to change crucial for developing an action plan to sustain the implementation of palliative care. The educational intervention was perceived by the managers to have improved the staff’s knowledge and provided opportunities for shared reflection. However, the managers still felt uncertain about how the spread of knowledge would continue within their organisations to reach all the staff. Managers emphasised their responsibility for the continuation of staff support in their daily work to maintain motivation and a positive attitude to change within the workforce. Managers also emphasised that staff members were given the opportunity to discuss difficult situations and reflect together on the content of care (Table 2).

#### Overcoming existing emotions

Managers described overcoming their initial frustration with the top-level manager´s decision to initiate the project alongside already ongoing initiatives after observing staff´s engagement with the education and willingness to implement palliative care. They also experienced that staff expressed fear and discomfort of palliative care and death, highlighting the need for continued managerial support during the implementation process.

We have assistant nurses here who have never experienced a death. I think it’s a lot of fear; it’s a fear of “How I’m going to handle and behave in these situations?” (Interview 8).

The managers highlighted the importance of overcoming resistance among staff for implementation of palliative care. Enthusiastic staff who inspired and motivated their colleagues were emphasised as a driving force that encouraged engagement and the spread of new ideas.

We have some enthusiasts; we have staff who are committed and who want to. If you get this group going, they can drive it forward a lot. That’s a good prerequisite. (Interview 18)

#### Reflection and dialogue as motivational strategies

The managers felt that successful implementation of palliative care depended on giving staff opportunities for reflection and discussion with their coworkers. Allowing staff to sit down, listen to each other and share experiences and perspectives strengthened relationships across professional categories and promoted and motivated the ongoing implementation. To further disseminate the knowledge, the managers hoped to assign ambassadors to lead discussions and promote an active, ongoing process to get everyone ʻon board’.

So, for those staff who have been participants in the education, it is positive. Yes, they spread good ‘ripples on the water’ to other units. (Interview 11)

The managers highlighted the value of staff training of difficult conversations in the educational seminars to be able to provide better palliative care when a resident was expected to die. These managers emphasised the need for reflection and proactive preparation to equip staff for difficult conversations.

To dare to have the difficult conversations, the tough conversations, you need to work preventively so that it doesn’t happen when you’re standing there in the situation that “Oh, now here comes a difficult conversation”. Instead, based on the older person or a relative or the culture or the background, you have the dialogue at an early stage in the working group on how to work. (Interview 15)

### Ripple benefits of education

The managers stated that the education contributed to increased knowledge of palliative care in general among staff, from early identification of palliative care needs to providing support at the end of life. Specifically, this training contributed to increased confidence, and safety in palliative care while fostering a common language, greater interprofessional understanding and improved collaboration. The education was seen as an important foundation for developing and implementing a more holistic palliative approach in nursing homes (Table 2).

#### Understanding the changes to the palliative approach

Following the educational intervention, the managers experienced a process of gradually implementing, understanding, reinforcing, and taking responsibility for establishing the palliative approach. The staff felt more grounded and secure when talking about death and gained a growing insight and a confirmation that palliative care was not solely about the end of life. Furthermore, the staff gained knowledge and awareness of patients’ customs and traditions before, during, and after death across different cultures and societies. The focus shifted towards promoting quality of life throughout the care process. This shift in the staff’s perspective to holistic palliative care contributed to a greater understanding of the importance of their daily nursing care.

I think that… if ‘everyone is on track,’ I can imagine that it will be good; I actually think so. Since they are already skilled, you know they are now thinking more about oral care, about turning the older person over and checking if they are clean, if they are calm and above all, checking that the patient is not in pain. (Interview 19)

The managers themselves were also inspired by the content of the education and were keen to continue implementing palliative care by using the practical advice provided, including the pain assessment scale. Managers highlighted that they had ‘a palliative care box’ at the nursing home containing practical items such as candles and sheets, and its content was revised after the education. The staff had been inspired to further develop and implement palliative care in the nursing home, for example, by creating a folder with routines and guidelines. Reflecting together during education helped managers become more aware of how staff members’ diverse backgrounds and past experiences shaped their approaches to palliative care. The education gave an opportunity for personal and professional development and increased the understanding of palliative care, which gradually became a natural part of the organisation’s culture. Overall, the managers felt that their participation in the education made them feel more confident in supporting staff in the relevant conversations.

It’s great that we actually sat down together to talk about this, and it’s the positive ambassadors who talking about the training in the coffee room – and it was so lovely. Now we’ve put together a little box in case someone passes away, and then someone else says, ‘Yes, that was a clever idea – we’ll do the same in our department too’. (Interview 22)

#### Cooperation between different professional groups

The managers observed that cross-domain cooperation among different professions had increased. They viewed interprofessional collaboration as a central component in implementing and sustaining palliative care in their organisations. However, the vision of breaking down barriers and collaborating more closely across professions was still evolving. The managers emphasised that this educational intervention had had a ripple effect but acknowledged the need for a much greater focus on communication and collaboration between the different professions to foster a greater understanding of each other’s roles and competencies.

Yes, when we had sick older persons who were on the verge of death, they (the staff) came together as a team and talked to each other, based on the same information from the education, they did a fantastic job on those occasions. (Interview 5)

Managers saw experienced staff members as essential role models who supported new staff in dealing with the emotional demands of providing palliative care. Managers were hopeful that increased interprofessional collaboration would support the continued implementation of this team-based care after the educational intervention ended. They also emphasised the need to strengthen teamwork, which had previously been lacking.

It went well, and it was good that there were different professional roles. I think it was really essential to be involved. The occupational therapist wasn’t with us much, but that became a discussion when we’ve been a whole team. (Interview 7)

## Discussion

Our qualitative follow-up study explored managers’ long-term experiences with the KUPA palliative care worksite training in nursing homes. Three major themes emerged from the analysis: navigating organisational barriers, empowering the action plan, and the ripple benefits of education. The managers encountered structural barriers, staff and manager instability, and staff overcoming emotional resistance to caring for dying residents. They perceived that staff’s increased knowledge, opportunities for shared reflection, and continued education facilitated palliative care. They also reported that the education provided a broader understanding of the palliative approach, from the early identification of palliative care needs to end-of-life support, while also strengthening collaboration within the team. This finding contrasts with the results from a pre-implementation study of the KUPA project [30], in which staff had difficulty identifying the early signs preceding death and viewed dying mainly as a happening, rather than a process.

The managers in this study expressed an understanding of the importance of participating in the education alongside staff, thereby promoting the implementation of a palliative approach in nursing homes. According to the managers, the palliative approach may be applied through early integration [31] when a person moves into a nursing home. However, despite managers being uniquely positioned to promote the implementation of knowledge-based care [15, 32–34], knowledge of the relationship between managerial roles and implementation outcomes remains nascent, as evidenced by a systematic review [32]. A key finding of the educational intervention in this study was that managers described the development of closer interprofessional collaboration over time, which fostered a greater understanding of one another’s roles and competencies. The education provided staff members from different professional backgrounds with a shared knowledge base and a common language for discussing palliative care. These findings are consistent with a previous qualitative evaluation of staff in the KUPA project [35], which showed that effective teamwork and collaboration in palliative care require ongoing development and a greater understanding of staff members’ diverse roles and skills [35]. Similar findings were also highlighted in a study of more than 1,000 nursing homes in Germany, where cooperation and education were identified as key components for improving palliative care in nursing homes [36]. Interprofessional collaboration is recognised as a critical component of effective palliative care [37]. To foster a positive team climate and full participation in the implementation of palliative care, team members need to overcome barriers such as competing interests [31]. Overall, our findings suggest that managers play an important role in recognising the contributions of all staff in providing palliative care and in supporting collaboration through communication.

Education and training were the most common implementation strategies for palliative care in long-term care facilities identified in a scoping review by Collingridge Moore and colleagues [38]. An internal assessment of the palliative care status at a nursing home before the start of an intervention may motivate staff to improve and may facilitate subsequent evaluation [38]. In the KUPA project, motivation was assessed based on the regional manager’s willingness to participate and their delegation to the front-line manager to continue the education, together with the inclusion criterion that the nursing home had no prior palliative care education. However, if the before-and-after assessment results [35, 39] had been shared with the staff, it may have further strengthened their motivation. Management enthusiasm for supporting staff involvement in improving palliative care was shown to be extremely important through the provision of protected time and resource allocation for participation in education and training [38]. However, in this study, staff shortages were a pressing concern for all managers, exacerbated more than in the previous study [10], because all staff were required to undergo palliative care training. Staff shortages in elderly care are a well-known problem in Sweden, requiring government intervention [9]. Involving older adults and their families in the implementation process may help them develop a better understanding of the aims and changes associated with palliative care. This could also make it easier to discuss treatment preferences with the family [38]. In the KUPA project, this strategy was not used; older adults and their families were included only as research participants [40, 41].

The incorporation of a palliative care intervention into day-to-day practice is characteristic of successful implementation [38]. This requires that the intervention be developed in collaboration with relevant representatives and pilot-tested so that it is adapted to both the target group and the context [31], as was the case for the KUPA intervention. Successful implementation strategies included reflection tasks related to staff’s practical care, incorporating a workbook for all seminars, and reference material that could be consulted as a training aid (link to Supplementary Material 1). These resources have also been shown to provide space for staff to talk openly about their feelings towards providing palliative care [38]. The selection of either internal or external facilitator is always an important part of implementation. Although internal facilitators understand the barriers to implementation within a specific facility and how to overcome them, they also hold dual roles and responsibilities. Conversely, external facilitators may have greater clarity regarding the facilitation role but often lack the context-specific knowledge needed to make necessary adaptations [38]. In the KUPA project, a combination of both approaches was used: initial guidance from external facilitators, followed by the continuation of the internal front-line manager.

There is a clear need for greater knowledge and planning regarding long-term strategies to sustain successful palliative care implementation. Existing literature provides limited guidance on long-term follow-up, ongoing staff retraining, funding mechanisms for continuous knowledge development, and approaches to maintaining enduring partnerships in palliative care. Consequently, sustainability considerations should be incorporated into implementation plans from the outset [38]. In this study, a key challenge to sustainability identified by managers was the recurring introduction of new directives from senior leadership, often driven by financial constraints [42]. These competing priorities forced managers to deprioritize staff competency development and slowed the pace of palliative care implementation to address more immediate organizational demands. Similar findings were reported in the process evaluation of the Palliative Care for Older People in Europe (PACE) training programme across seven European countries, which documented interruptions in implementation [43–45]. Managers who continued the work often cited its perceived value to clinical practice [44], consistent with the theme of “Loss of Momentum” identified in the current study. However, additional barriers emerged, including inadequate transfer of information to newly appointed managers and the impact of unexpected workplace challenges that hindered implementation efforts. Previous research has demonstrated that support from senior leadership can strengthen commitment among managers and staff by clearly communicating organizational priorities and providing essential resources, such as policies, staffing, training opportunities, and funding [32, 46]. Such support has been shown to enhance an organization’s capacity for change [47], although this factor was not specifically examined in the present follow-up study. Comparison with findings from our earlier study conducted one year prior [10] revealed both continuity and change in implementation barriers. Persistent challenges included staff shortages and limited financial resources, which restricted the time available for staff to engage in activities aimed at improving palliative care. In contrast, barriers related to staff members’ lack of confidence in caring for dying individuals had diminished over time. New challenges emerged, however, including instability in management positions and uncertainty among managers regarding responsibility for sustaining implementation efforts. These findings underscore the importance of understanding barriers to sustainability, as the literature highlights their critical role in informing intervention development, supporting effective implementation, and guiding decisions related to programme continuation, adaptation, scaling, and broader dissemination [48–51].

### Limitations

Five of the 20 front-line managers (25%) had changed positions to another nursing home by the time the data were collected, creating greater uncertainty regarding the events and decisions that occurred during implementation. This leadership instability is concerned to have adversely affected the richness of these five interviews. Furthermore, during the interviews, some managers expressed uncertainty regarding plans for the continuation of palliative care implementation. One possible explanation is that managers with an educational background in social work need more support before taking full responsibility for continuing the implementation of a palliative care approach, an issue addressed previously [16]. The educational background of facilitating managers needs to be considered in future implementations. Finally, a limitation of the KUPA project is that staff attendance at each seminar was recorded in only half of the nursing homes, which limits the ability to assess whether attendance rates affected progress towards the goal of implementing palliative care.

## Conclusions

Our long-term follow-up implementation study shows a broader view of palliative care as a process from early signs to end-of-life was applied in daily nursing care. Managers perceived staff as more confident in meeting dying people after the implementation. They described that a closer interprofessional collaboration developed over time, fostering a greater understanding of each other’s roles and competencies. The finding also revealed that implementing palliative care in nursing homes was a demanding and complex issue for managers. The perception of barriers changed during the implementation process. Staff shortage contributed to the pressure, and work-related instability appeared due to turbulence and high staff turnover, which was challenging to handle for the manager. From the leadership perspective, the most important lesson from this study is that a successful implementation of palliative care requires a long-term commitment, financial resources, a supportive organisation, and manager competence.

## Supplementary Information

Supplementary Material 1. Benzein and Ahlström 2019. The booklet themed meetings about palliative care within health care. Supplement in: Bökberg et al. 2019 Evaluation of person-centeredness in nursing homes after a palliative care intervention: pre- and post-test experimental design. BMC Palliative Care (2019) 18:44. https://doi.org/10.1186/s12904-019-0431-8 .

## Data Availability

The dataset used and analysed during the study is available from the project leader (G.A.) upon written request and in accordance with ethical approval.
